# Supplementary material for: Anomalous spin current anisotropy in a noncollinear antiferromagnet
Source: Nat Commun. 2023 Sep 21;14:5873. doi: 10.1038/s41467-023-41568-0 (PMC10514083; doi:10.1038/s41467-023-41568-0)
Supplement: Supplementary file 1 — Supplementary Information [file 41467_2023_41568_MOESM1_ESM.pdf]

Supporting Information

Anomalous spin current anisotropy in a noncollinear antiferromagnet

Cuimei Cao[1a], Shiwei Chen[2,6a], Rui-Chun Xiao[3], Zengtai Zhu[4,5], Guoqiang Yu[4,5], Yangping Wang[1], Xuepeng Qiu[6], Liang Liu[7,8], Tieyang Zhao[8], Ding-Fu Shao[9,*], Yang Xu[1,*], Jingsheng Chen[8,*], and Qingfeng Zhan[1,*]

[1]*Key Laboratory of Polar Materials and Devices (MOE), School of Physics and Electronic Science, East China Normal University, Shanghai 200241, People's Republic of China*

[2]*School of Physics, Hubei University, Wuhan 430062, People's Republic of China*

[3]*Institute of Physical Science and Information Technology, Anhui University, Hefei 230601, People's Republic of China*

[4]*Songshan Lake Materials Laboratory, Dongguan, Guangdong 523808, People's Republic of China*

[5]*Beijing National Laboratory for Condensed Matter, Physics Institute of Physics, Chinese Academy of Sciences, Beijing 100190, People's Republic of China*

[6]*Shanghai Key Laboratory of Special Artificial Microstructure Materials, School of Physics Science and Engineering, Tongji University, Shanghai 200092, People's Republic of China*

[7]*Key Laboratory of Artificial Structures and Quantum Control (Ministry of Education), School of Physics and Astronomy, Shanghai Jiao Tong University, Shanghai, 200240, China*

[8]*Department of Materials Science and Engineering, National University of Singapore, Singapore, Singapore*

[9]*Key Laboratory of Materials Physics, Institute of Solid State Physics, HFIPS, Chinese Academy of Sciences, Hefei 230031, People's Republic of China*

[a)] C. Cao and S. Chen contributed equally to this work.

[*] Authors to whom correspondence should be addressed: dfshao@issp.ac.cn, yxu@phy.ecnu.edu.cn, msecj@nus.edu.sg, qfzhan@phy.ecnu.edu.cn

Contents:

Supplementary Note 1: Spin Hall conductivity tensors of noncollinear antiferromagnetic Mn_3Pt

Supplementary Note 2: Characterization of the Mn_3Pt thin film

Supplementary Note 3: Spin-torque ferromagnetic resonance (ST-FMR) measurements in the Mn_3Pt/Py and Pt/Py bilayers

Supplementary Note 4: ST-FMR measurements in the Mn_3Pt/Py bilayer

Supplementary Note 5: Calculation of the contribution from the Oersted field to the field-like-torque A_{FL}^{y}

Supplementary Note 6: The exchange bias effect of Mn_3Pt based heterostructures

Supplementary Note 7: The anomalous Hall effect at different ϕ_E

Supplementary Note 8: The AHE loop shift of the Mn_3Pt/Ti/CoFeB/MgO/SiO_2 heterostructure with ϕ_E = 45 °

Supplementary Note 9: The AHE loops in the Mn_3Pt/Ti/CoFeB/MgO/SiO_2 heterostructure under I = $\pm$20 mA for different ϕ_E

Supplementary Note 10: Evaluation of Joule heating effect

Supplementary Note 11: Field-free current-induced magnetization switching at different ϕ_E

Supplementary Note 12: The AHE loops in the Mn_3Pt/Ti/CoFeB/MgO/SiO_2 heterostructure for different premagnetization field

Note 1. Spin Hall conductivity tensors of noncollinear antiferromagnetic Mn_3Pt

Table S1 shows the tensors of time reversal-even ($\mathcal{T}$-even) and time reversal-odd ($\mathcal{T}$-odd) spin Hall conductivity (SHC) in noncollinear antiferromagnetic Mn_3X (X= Pt, Ir or Rh) for $\phi_E = 0°$[1]. For a finite ϕ_E, the coordinate system is transformed by a rotation matrix D, and the associated SHC tensors are then obtained as follows

$$\sigma_{(\phi_E)}{}_{i,j}^{s,k} = \sum_{l,m,n} D_{il} D_{jm} D_{kn}\, \sigma_{(\phi_E=0°)}{}_{lm}^{s,n}.$$

For the SHC components associated with the out-of-plane spin current investigated in this work, we find the $\mathcal{T}$-even SHC are dependent on the current direction as:

$$\sigma_{zx}^{x,\text{even}}(\phi_E) = b\cos 2\phi_E,$$

$$\sigma_{zx}^{y,\text{even}}(\phi_E) = -d + b\sin 2\phi_E,$$

$$\sigma_{zx}^{z,\text{even}}(\phi_E) = a(\sin\phi_E - \cos\phi_E). \quad \text{(S1)}$$

Therefore, the magnitudes of the $\mathcal{T}$-even SHC are the same for currents along the primary directions [100] ($\phi_E = 0°$) and [010] ($\phi_E = 90°$).

Similarly, we can obtain the ϕ_E dependence of $\mathcal{T}$-odd SHC:

$$\sigma_{zx}^{x,\text{odd}}(\phi_E) = B + D\sin 2\phi_E,$$

$$\sigma_{zx}^{y,\text{odd}}(\phi_E) = D\cos 2\phi_E,$$

$$\sigma_{zx}^{z,\text{odd}}(\phi_E) = A(\sin\phi_E + \cos\phi_E). \quad \text{(S2)}$$

These $\mathcal{T}$-odd SHC for currents along the primary directions [100] ($\phi_E = 0°$) and [010] ($\phi_E = 90°$) also have the same magnitudes.

However, the net SHC σ_{zx}^{p} contributed by the intertwined $\mathcal{T}$-even and $\mathcal{T}$-odd spin Hall effect (SHE) has a more complicated anisotropy in Mn_3X, as shown in Eq. (3) in the main text. We schematically plot the angular dependence of σ_{zx}^{p}, $\sigma_{zx}^{p,\text{odd}}$, $\sigma_{zx}^{p,\text{even}}$ in Fig. 1e by assuming the $\mathcal{T}$-odd SHE is stronger than $\mathcal{T}$-even SHE using the parameters of $A = 1$, $B = 0.5$, $D = 1$, and $a = 0.5$, $b = 0.5$, $d = -0.5$.

Table S1: The tensors for $\mathcal{T}$-even and $\mathcal{T}$-odd SHC in noncollinear antiferromagnetic Mn_3X.

$\boldsymbol{\phi_E = 0°}$ ($\boldsymbol{x} \parallel [\mathbf{100}]$, $\boldsymbol{y} \parallel [\mathbf{010}]$, $\boldsymbol{z} \parallel [\mathbf{001}]$)	$\boldsymbol{\sigma^x} = \begin{bmatrix} \boldsymbol{\sigma^x_{xx}} & \boldsymbol{\sigma^x_{xy}} & \boldsymbol{\sigma^x_{xz}} \\ \boldsymbol{\sigma^x_{yx}} & \boldsymbol{\sigma^x_{yy}} & \boldsymbol{\sigma^x_{yz}} \\ \boldsymbol{\sigma^x_{zx}} & \boldsymbol{\sigma^x_{zy}} & \boldsymbol{\sigma^x_{zz}} \end{bmatrix}$	$\boldsymbol{\sigma^y} = \begin{bmatrix} \boldsymbol{\sigma^y_{xx}} & \boldsymbol{\sigma^y_{xy}} & \boldsymbol{\sigma^y_{xz}} \\ \boldsymbol{\sigma^y_{yx}} & \boldsymbol{\sigma^y_{yy}} & \boldsymbol{\sigma^y_{yz}} \\ \boldsymbol{\sigma^y_{zx}} & \boldsymbol{\sigma^y_{zy}} & \boldsymbol{\sigma^y_{zz}} \end{bmatrix}$	$\boldsymbol{\sigma^z} = \begin{bmatrix} \boldsymbol{\sigma^z_{xx}} & \boldsymbol{\sigma^z_{xy}} & \boldsymbol{\sigma^z_{xz}} \\ \boldsymbol{\sigma^z_{yx}} & \boldsymbol{\sigma^z_{yy}} & \boldsymbol{\sigma^z_{yz}} \\ \boldsymbol{\sigma^z_{zx}} & \boldsymbol{\sigma^z_{zy}} & \boldsymbol{\sigma^z_{zz}} \end{bmatrix}$
$\boldsymbol{\mathcal{T}}$-odd	$\begin{bmatrix} E & A & A \\ B & C & D \\ B & D & C \end{bmatrix}$	$\begin{bmatrix} C & B & D \\ A & E & A \\ D & B & C \end{bmatrix}$	$\begin{bmatrix} C & D & B \\ D & C & B \\ A & A & E \end{bmatrix}$
$\boldsymbol{\mathcal{T}}$-even	$\begin{bmatrix} 0 & -a & a \\ b & -c & -d \\ -b & d & c \end{bmatrix}$	$\begin{bmatrix} c & -b & D \\ a & 0 & -a \\ -d & b & -c \end{bmatrix}$	$\begin{bmatrix} -c & -d & b \\ d & c & -b \\ -a & a & 0 \end{bmatrix}$

Note 2. Characterization of the Mn_3Pt thin film

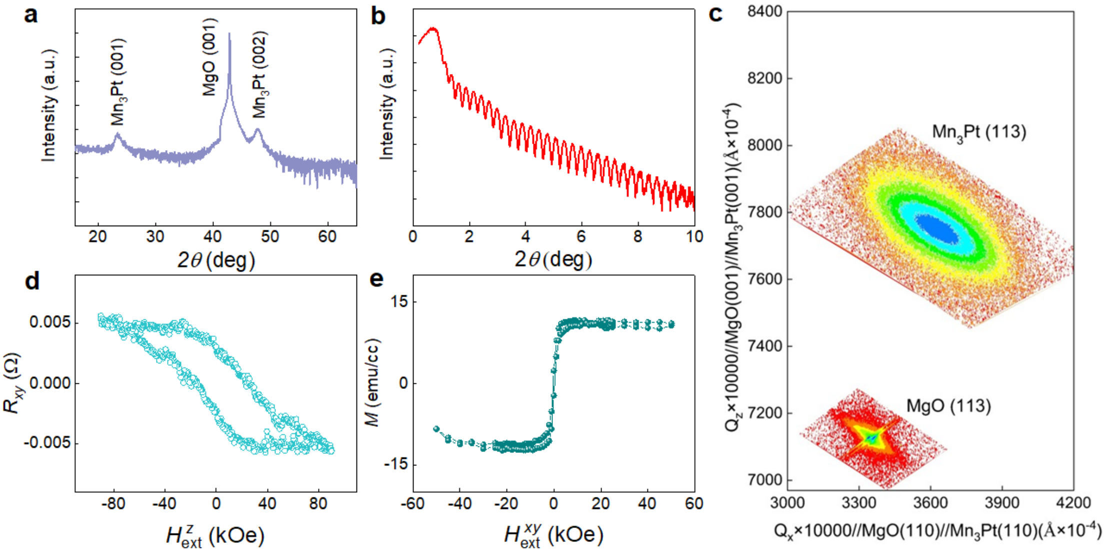

Fig. S1 Crystal structure characterization and the magnetic property of Mn_3Pt. **a** The X-ray diffraction θ-2θ scan pattern of a Mn_3Pt(12 nm) film deposited on the MgO(001) substrate. **b** The X-ray reflectivity spectrum. **c**, Reciprocal space mapping around the (113) reflection. **d** The Hall resistance R_{xy} versus the out-of-plane magnetic field H^z_{ext}. **e** The in-plane magnetic hysteresis loop measured at room temperature.

The crystalline quality of the Mn_3Pt film was checked by using the X-ray diffraction technique. A representative θ-2θ pattern is shown in Fig. S1a. Except for the (001) diffraction peak from the MgO substrate, the presence of peaks in the (001) family from Mn_3Pt illustrates the high degree of (001) texture. Figure S1b shows the X-ray reflectivity spectrum of an as-grown MgO(001)/Mn_3Pt heterostructure. Multiple oscillations can be seen up to a high 2θ angle, indicating a smooth and uniform film surface. Figure S1c shows the typical reciprocal space mapping pattern of the (113) plane. The presence of only the (113) peak is consistent with the characteristic of the Mn_3Pt crystal structure, which indicates that Mn_3Pt film grew epitaxially on the MgO substrate. Figure S1d shows the Hall resistance (R_{xy}) as a function of the out-of-plane magnetic field (H^z_{ext}) of the MgO(001)/Mn_3Pt heterostructure at room temperature. The obvious anomalous Hall effect confirms the noncolinear AFM nature of Mn_3Pt. The in-plane magnetic hysteresis loop was measured using a magnetic property measurement

system (MPMS, Quantum Design) with a 50 kOe in-plane magnetic field applied, as shown in Fig. S1e. The saturation magnetization M_s ~ 10 emu/cm^3 can be obtained after subtracting the background signal, comparable to the reported value[2].

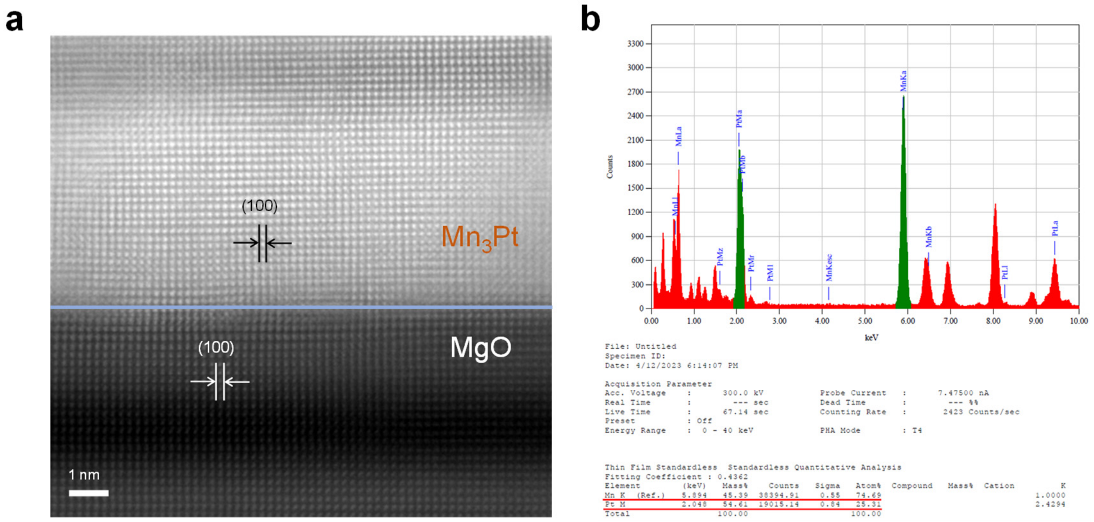

Fig. S2 Epitaxial structure characterization and atomic ratio of Mn_3Pt. **a** The cross-sectional transmission electron microscopy image and **b** the atomic ratio measured by EDS of a Mn_3Pt(12 nm) film deposited on the MgO(001) substrate.

The cross-sectional crystalline structure of the Mn_3Pt layer on the MgO (001) substrate imaged by aberration-corrected scanning transmission electron microscopy (AC-STEM, FEI Titan Themis 200) operated at 200 kV is shown in Fig. S2a. Good epitaxial growth of the Mn_3Pt thin film with a sharp interface to the MgO substrate can be observed. A Mn_3Pt unit cell along the [100] direction matches well with a MgO unit cell along the [100] direction. Furthermore, the atomic ratio of our sample has been checked by energy-dispersive X-ray spectroscopy (EDS). As shown in Fig. S2b, the atomic ratio is determined to be Mn : Pt = 74.69 : 25.31 ≈ 3 : 1.

Note 3. Spin-torque ferromagnetic resonance (ST-FMR) measurements in the Mn_3Pt/Py and Pt/Py bilayers

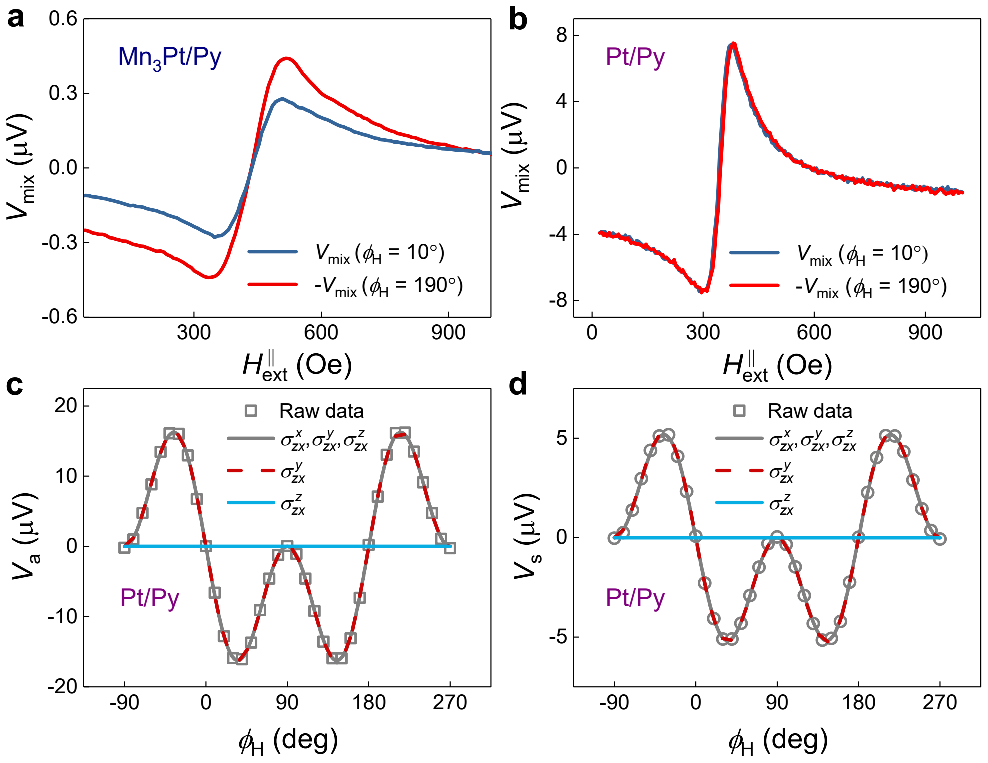

Fig. S3 The ST-FMR signals of the Mn_3Pt/Py and Pt/Py samples. Typical ST-FMR signal of the (**a**) Mn_3Pt(12 nm)/Py (13 nm) and (**b**) Pt(6 nm)/Py(6 nm) samples, with the magnetization oriented at 10° (blue line) and 190° (red line) relative to the current direction. **c** The antisymmetric component V_a (hollow squares for the raw data and the grey line for fitting) of Pt/Py bilayer as a function of the angle between the magnetic field and current ϕ_H and its decomposition into contributions from the spin polarizations σ^p_{ij}, where i, j, and p are the generated spin-current, the driven charge-current, and spin polarization directions, respectively. **d** Similar to (**c**) for the symmetric component V_s.

In the ST-FMR measurements, the magnetic moments in Py are set into precession by the SOT associated with the spin current generated in Mn_3Pt. Combined with the anisotropic magnetoresistance of Py, the output signal is a rectified voltage V_{mix}, whose lineshape can be decomposed into a symmetric component V_s and an antisymmetric component V_a near the resonant condition[3-5]:

$$V_{mix} = V_s \frac{\Delta H^2}{\Delta H^2 + \left(H^{\parallel}_{ext} - H_0\right)^2} + V_a \frac{\Delta H\left(H^{\parallel}_{ext} - H_0\right)}{\Delta H^2 + \left(H^{\parallel}_{ext} - H_0\right)^2}, \quad \text{(S3)}$$

where H_0 is the resonant field, and ΔH is the linewidth of the resonant peak. The

components V_s and V_a characterize the in-plane SOT $\tau_{\parallel}$ and out-of-plane SOT $\tau_{\perp}$, respectively, allowing the full determination of the damping-like SOT $\mathbf{m} \times (\mathbf{m} \times \mathbf{p})$ and the field-like SOT $\mathbf{m} \times \mathbf{p}$ from all possible spin polarizations p ($p = x, y, z$)[6,7]. Note that considering the in-plane $\mathbf{m}$ of Py, $\tau_{\perp}$ may come from the damping-like SOT associated with σ_{zx}^{z}, while it also includes the field-like contribution from σ_{zx}^{y}, σ_{zx}^{x}, and the Oersted field.

Figure S3a shows the representative ST-FMR spectra for the Mn_3Pt/Py bilayer which measured at 7 GHz with ϕ_H = 10° and 190°, respectively, which does accord with the ST-FMR signals obtained at negative and positive magnetic fields. Here, ϕ_H is the angle between the microwave current I_{rf} and the magnetic field $H_{ext}^{\parallel}$. The observation of the ST-FMR signals V_{mix} does not exhibit a perfect inversion with the reversal of $H_{ext}^{\parallel}$, i.e., V_{mix} ($\phi_H =$ 10°) and $-V_{mix}$ ($\phi_H =$ 190°) do not overlap, providing strong evidence of the presence of an unconventional SOT derived from spin polarizations other than σ_{zx}^{y}[2,8-13].

In a typical heavy metal/ferromagnet bilayer with no broken crystal or magnetic structure symmetry like Pt/Py, the current-induced spin torque mainly comes from the Oersted field, the SHE, and/or the Rashba-Edelstein effect, which all exhibit a $\cos\phi_H \sin 2\phi_H$ dependence[3]. Different from the case for Mn_3Pt/Py bilayer, as shown in Fig. S3b, the ST-FMR signals V_{mix} for the Pt/Py bilayer exhibit a perfect inversion with the reversal of $H_{ext}^{\parallel}$. This is indeed the case for Pt/Py, as shown in Figs. S3c and 3d, pointing to no extra contribution except for σ_{zx}^{y} from the conventional SHE and confirming the reliability of our ST-FMR measurements.

Note 4. ST-FMR measurements in the Mn3Pt/Py bilayer

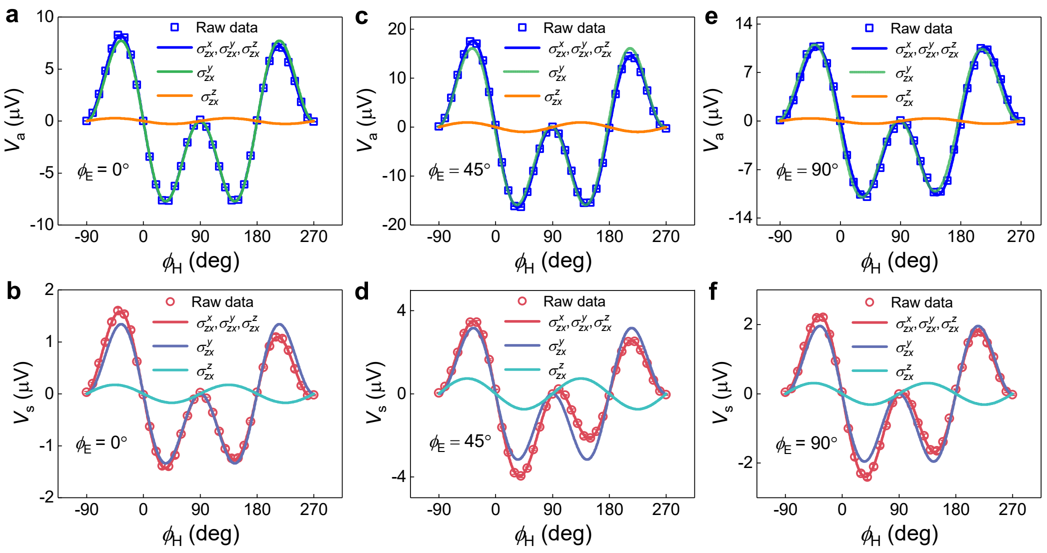

Fig. S4 Characterization of the SOT associated with σ_{ij}^{p} ($p = x, y, z$) by ST-FMR in Mn3Pt/Py. a-f The ϕ_{H} dependence of V_{a} and V_{s} at selected ϕ_{E} of 0°, 45°, and 90°, here ϕ_{E} is the angle between the microwave current and the [100] direction of Mn3Pt. Their decomposition into contributions from the spin polarizations σ_{ij}^{p} can be derived from fittings (solid lines) to Eqs. (4) and (5) in the main text.

To examine the torque components quantitatively, the ST-FMR measurements as a function of the in-plane magnetic field angle ϕ_{H} were performed. Figures S4a-4f shows the ϕ_{H} dependence of V_{a} and V_{s} at selected ϕ_{E} of 0°, 45°, and 90°, where V_{a} and V_{s} can be well fitted by adding additional, unconventional torque terms through Eqs. (4) and (5) in the main text, respectively. These results give a strong evident, apart from σ_{zx}^{y}, that the presence of nonzero σ_{zx}^{x} and σ_{zx}^{z} in Mn3Pt/Pt bilayer. Moreover, the σ_{zx}^{z} contribution is enhanced for $\phi_{\mathrm{E}} = 45°$ by comparing with $\phi_{\mathrm{E}} = 0°$, which is consistent with the previous observation of a stronger (weaker) σ_{zx}^{z} for current I applied parallel (perpendicular) to the magnetic mirror plane of Mn3Pt[2]. More importantly, an obvious discrepancy can be found between the cases of $\phi_{\mathrm{E}} = 0°$ and $\phi_{\mathrm{E}} = 90°$, which is unexpected considering their equivalency in both the crystal and magnetic structures, especially for high symmetry (001) plane of the spin source material with cubic structure.

Note 5. Calculation of the contribution from the Oersted field to the field-like-torque A_{FL}^{y}

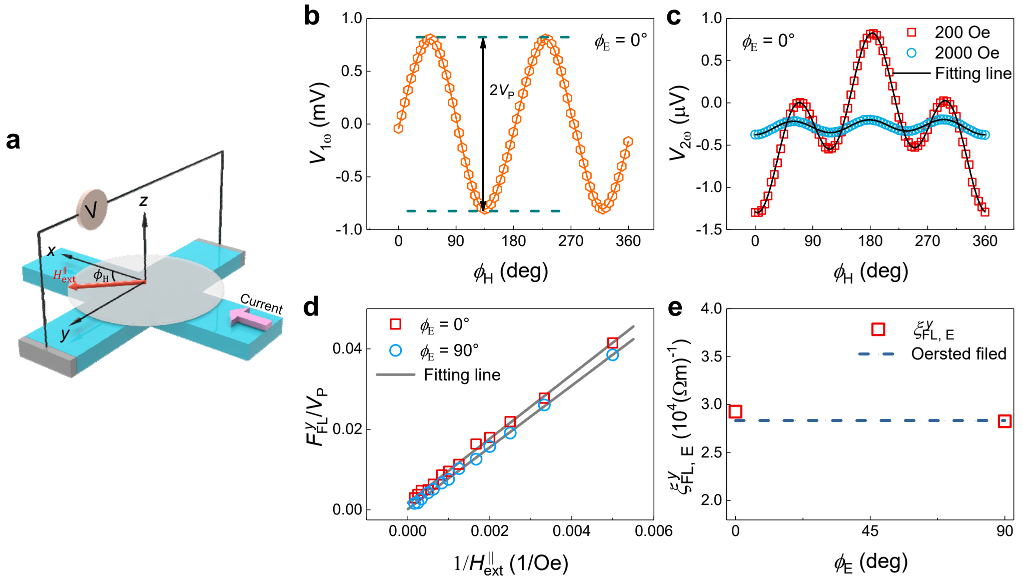

Fig. S5 a Geometry of the harmonic Hall measurements with rotating magnetic field in the *xy* plane $H_{\mathrm{ext}}^{\parallel}$. **b** Typical first harmonic Hall voltage as a function of ϕ_{H} under $H_{\mathrm{ext}}^{\parallel}$ = 200 Oe and an a.c. current along the [100] direction (ϕ_{E} = 0°). **c** Typical second harmonic Hall voltage as a function of ϕ_{H} for an a.c. current along the [100] direction (ϕ_{E} = 0°) for two different magnitudes of magnetic field. **d** Normalized second harmonic Hall voltages for ϕ_{E} = 0° and 90° as a function of the inverse of the applied magnetic field. **e** Symbols: measured values of $\xi_{\mathrm{FL,E}}^{y}$ from the second harmonic Hall measurements. Dashed line: estimated contribution from the Oersted field.

According to the previous work[13], we can use the value of field-like-torque A_{FL}^{y} generated by σ_{zx}^{y} as a measure of the current density in the heavy metal to quantify the amplitude of the damping-like torque efficiencies per unit current density only if the Oersted field is the dominant source for A_{FL}^{y}. Therefore, in order to investigate whether the Oersted field is the dominant source for A_{FL}^{y} in our samples, we should calculate the A_{FL}^{y} by second harmonic Hall measurement.

A sinusoidal a.c. current with a frequency of 13.7 Hz was applied in a Hall bar device with ϕ_{E} = 0° and 90°, and the first and second harmonic Hall voltages were

simultaneously measured by two lock-in amplifiers. An in-plane magnetic field $H_{\text{ext}}^{\parallel}$ was applied at various angles ϕ_{H} relative to the current direction, as shown in Fig. S5a. The a.c. current through the Mn_3Pt layer produces both periodic in-plane and out-of-plane torques on the magnetization of the Py layer, causing magnetization oscillation around the equilibrium direction that changes both the anomalous Hall resistance and the planar Hall resistance[14]. The first ($V_{1\omega}$) and second harmonic Hall voltage $V_{2\omega}$, is then[14-17]:

$$V_{1\omega} = V_{\text{P}} \sin 2\phi_{\text{H}} \sin^2 \theta + V_{\text{A}} \cos \theta, \tag{S4}$$

$$V_{2\omega} = D_{\text{DL}}^{y} \cos \phi_{\text{H}} + D_{\text{DL}}^{x} \sin \phi_{\text{H}} + D_{\text{DL}}^{z} \cos 2\phi_{\text{H}} + F_{\text{FL}}^{y} \cos \phi_{\text{H}} \cos 2\phi_{\text{H}} + F_{\text{FL}}^{x} \sin \phi_{\text{H}} \cos 2\phi_{\text{H}} + F_{\text{FL}}^{z}, \tag{S5}$$

with

$$D_{\text{DL}}^{y} = -\frac{\tau_{\text{DL}}^{y}}{\gamma} \frac{V_{\text{A}}}{2(H_{\text{ext}}^{\parallel} + H_{\text{k}})} + V_{\text{ANE}} + V_{\text{ONE}} H_{\text{ext}}^{\parallel}, \tag{S6}$$

$$D_{\text{DL}}^{x} = -\frac{\tau_{\text{DL}}^{x}}{\gamma} \frac{V_{\text{A}}}{2(H_{\text{ext}}^{\parallel} + H_{\text{k}})}, \tag{S7}$$

$$D_{\text{DL}}^{z} = -\frac{\tau_{\text{DL}}^{z}}{\gamma} \frac{V_{\text{P}}}{H_{\text{ext}}^{\parallel}}, \tag{S8}$$

$$F_{\text{FL}}^{y} = -H_{\text{FL}}^{y} \frac{V_{\text{P}}}{H_{\text{ext}}^{\parallel}}, \tag{S9}$$

$$F_{\text{FL}}^{x} = -H_{\text{FL}}^{x} \frac{V_{\text{P}}}{H_{\text{ext}}^{\parallel}}, \tag{S10}$$

$$F_{\text{FL}}^{z} = H_{\text{FL}}^{z} \frac{V_{\text{A}}}{2(H_{\text{ext}}^{\parallel} + H_{\text{k}})} + C, \tag{S11}$$

where D_{DL}^{p} and F_{DL}^{p} are coefficients for the damping-like and field-like SOT generated by σ_{zx}^{p}, respectively. τ_{DL}^{p} and H_{FL}^{p} are the damping-like torques and the effective fields associated with the field-like torque generated by σ_{zx}^{p}, respectively. Note that the in-plane Oersted field as well as the spin-transfer torque can contribute to H_{FL}^{y}. θ and ϕ_{H} are the polar and azimuthal angles of the magnetization vector, respectively. V_{P} is the coefficient of the planar Hall voltage, $V_{\text{PHE}} = V_{\text{P}} \sin 2\phi_{\text{H}}$, for a given direction the alternating current, and V_{A} is the coefficient of the anomalous Hall voltage $V_{\text{AHE}} = V_{\text{A}} \cos \theta$. Note that the contribution of V_{AHE} to $V_{1\omega}$ is negligible since the Py layer exhibits an in-plane anisotropy. V_{ANE} is an anomalous

Nernst voltage. $V_{\mathrm{ONE}} H_{\mathrm{ext}}^{\parallel}$ is a voltage due to the ordinary Nernst effect. H_{k} characterizes the out-of-plane magnetic anisotropy (positive for samples with an in-plane anisotropy), and C accounts for a constant background. The field-like torque efficiencies per unit electric field associated with each component p are

$$\xi_{\mathrm{FL,E}}^{p} = \frac{2e}{\hbar} M_{\mathrm{s}} t_{\mathrm{FM}} \frac{\mu_0 H_{\mathrm{FL}}^{p}}{E}, \tag{S12}$$

where E is the peak electric field associated with the a.c. current.

We show the ϕ_{H} dependence for the harmonic Hall voltages of the Mn_3Pt(12 nm)/Py(13 nm) sample in Fig. S5b-5c for an a.c. current applied in the [100] direction (ϕ_{E} = 0°). In Fig. S5c, we show the second harmonic Hall voltage as a function of ϕ_{H} under an 200 Oe (red) and 2000 Oe (blue) magnetic field. The corresponding fitting lines based on Eq. (S5) are shown in black lines. To quantify the effective fields for the out-of-plane torque terms, F_{FL}^{y} is normalized with respect to V_{P}, plotted as a function of $1/H_{\mathrm{ext}}^{\parallel}$ which is fitted to straight lines for ϕ_{E} = 0°, as shown in Fig. S5d. From the slopes of the fitting lines and Eq. (S12), we calculate the field-like torque efficiencies $\xi_{\mathrm{FL,E}}^{y}$ and show the corresponding results for ϕ_{E} = 0° and 90° in Fig. S5e. It is found that $\xi_{\mathrm{FL,E}}^{y}$ shows little variation with ϕ_{E}. Meanwhile, the current-induced Oersted field is $H_{\mathrm{Oe}} = \frac{1}{2} j_{\mathrm{HM}} t_{\mathrm{HM}}$, where j_{HM} and t_{HM} are the current density and thickness of the Mn_3Pt layer, and the Oersted field translates into a torque efficiency as following:

$$\xi_{\mathrm{Oersted,E}}^{y} = \frac{2e}{\hbar} \frac{\mu_0 M_{\mathrm{s}} t_{\mathrm{FM}} t_{\mathrm{HM}}}{2\rho_{xx}}, \tag{S13}$$

where ρ_{xx} is the resistivity of the Mn_3Pt layer. Substituting in corresponding values of our samples, $\mu_0 M_{\mathrm{s}}$ = 0.79 T, t_{FM} = 13 nm, t_{HM} = 12 nm, and ρ_{xx} = 176 μΩcm[18] yields the blue dashed line in Fig. S5e, in good agreement with the data points. The Oersted torque is seen to contribute at least 96% of the field-like torque efficiency $\xi_{\mathrm{FL,E}}^{y}$ regardless of the electric-field angle ϕ_{E}, indicating A_{FL}^{y} is dominated by the Oersted field, making A_{FL}^{y} a good measure of the current density in the Mn_3Pt layer. A similar

approach was applied in a previous study of RuO_2/Py bilayer[13].

Note 6. The exchange bias effect of Mn_3Pt based heterostructures

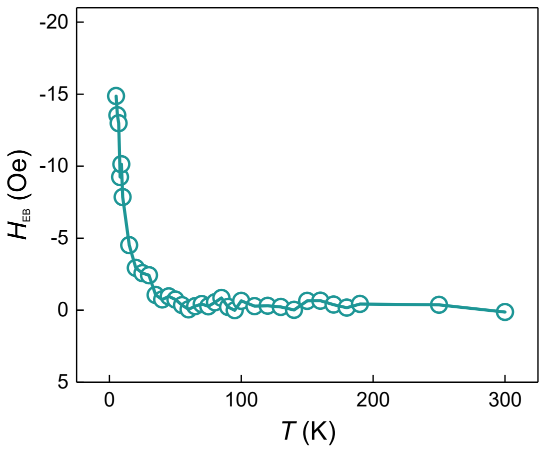

Fig. S6 The temperature dependence of the exchange bias field of the Mn_3Pt/Ti/CoFeB/MgO/SiO_2 heterostructure extracted from the AHE curves.

To determine the influence of the exchange bias on the field-free magnetization switching of the Mn_3Pt/Ti/CoFeB/MgO/SiO_2 heterostructure, we measured the AHE curves in the temperature range of 5-300 K. The values of the exchange bias field H_{EB} extracted from AHE curves at different temperatures are shown in Fig. S6. No exchange bias effect was observed in the wide temperature range of 100 to 300 K, which can be attributed to the thick Ti layer which effectively decouples the antiferromagnetic Mn_3Pt and ferromagnetic CoFeB layer. The exchange bias effect was only observed at sufficiently low temperatures (below ~50 K) with enhanced exchange coupling. This result unambiguously excludes the exchange bias as the possible reason for the field-free switching behavior of Mn_3Pt/Ti/CoFeB/MgO/SiO_2 heterostructure at room temperature.

Note 7. The anomalous Hall effect at different ϕ_E

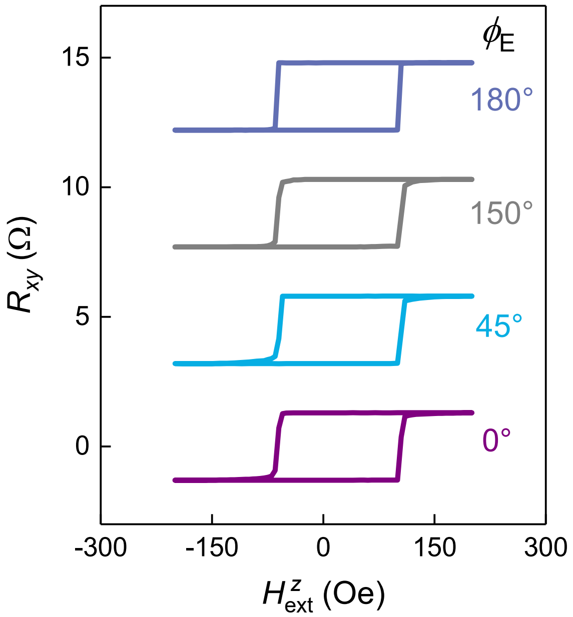

Fig. S7 The anomalous Hall resistance R_{xy} as a function of the out-of-plane magnetic field H_{ext}^z at different ϕ_E (0°, 45°, 150° and 180°).

As shown in Fig. S7, the anomalous Hall effect (AHE) curves of Mn_3Pt/Ti/CoFeB/MgO/SiO_2 heterostructure at different ϕ_E (0°, 45°, 150°, and 180°) were measured under the magnetic field along the *z* axis. According to the square shape of the out-of-plane hysteresis, we identified the good perpendicular magnetic anisotropy of the sample.

Note 8. The AHE loop shift of the Mn_3Pt/Ti/CoFeB/MgO/SiO_2 heterostructure with ϕ_E = 45 °

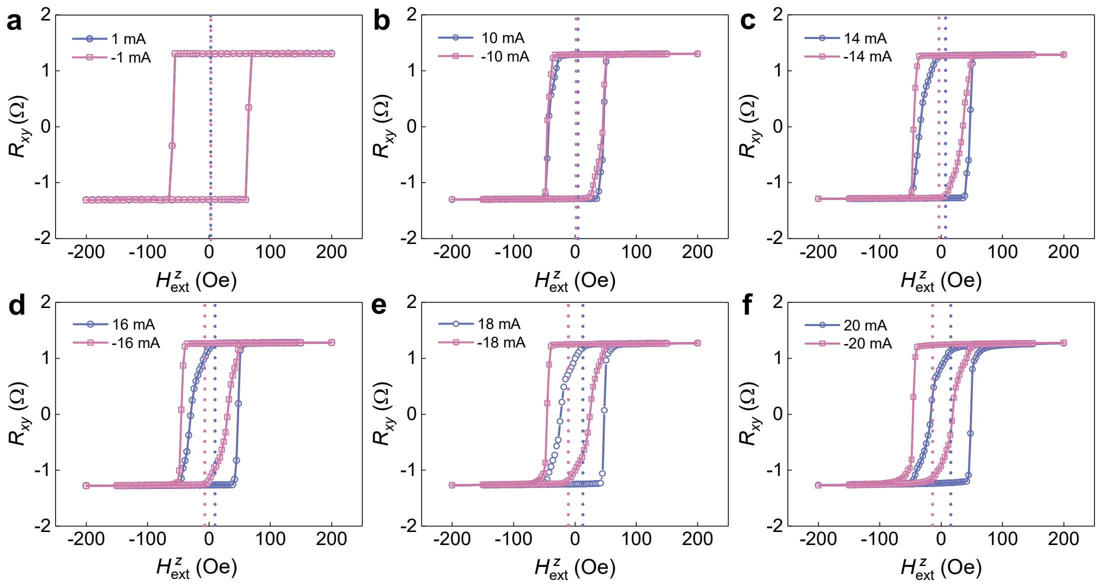

Fig. S8 The anomalous Hall loops under different pulsed d.c. currents for the Hall bar device with ϕ_E = 45°.

Figure S8 shows the evolution of the AHE loop shift with increasing the pulsed d.c. current in the Mn_3Pt/Ti/CoFeB/MgO/SiO_2 heterostructure for Hall bar device with ϕ_E = 45°. As shown in Figs. S8a and 8b, there is no shift observed when the current amplitude is below 10 mA. While above 10 mA, in Figs. S8c-8f, AHE loop shift occurs and increases almost linearly with the increase of I, indicating the existence of H^z_{eff}.

Note 9. The AHE loops in the Mn_3Pt/Ti/CoFeB/MgO/SiO_2 heterostructure under $I = \pm 20$ mA for different ϕ_E

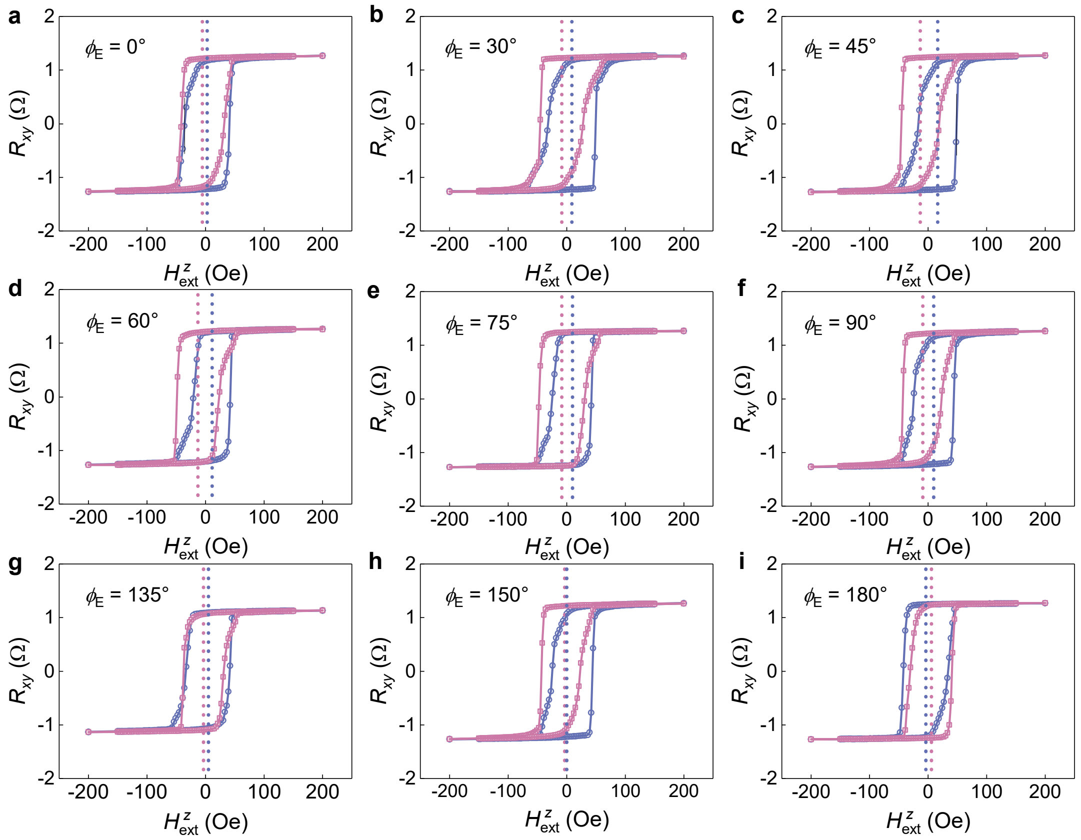

Fig. S9 AHE loops under a pulsed d.c. current $I = \pm 20$ mA for varying ϕ_E.

Figure S9 shows the AHE loops of the Mn_3Pt/Ti/CoFeB/MgO/SiO_2 heterostructure measured by applying a d.c. current $I = \pm 20$ mA for different ϕ_E ranging from 0° to 180°. The values of the AHE loop shift vary with ϕ_E, indicating the anisotropy of H^z_{eff} which is induced by the anisotropy of σ^z_{zx}. The ϕ_E dependence of H^z_{eff} obtained is summarized in the main text (Fig. 3e).

Note 10. Evaluation of Joule heating effect

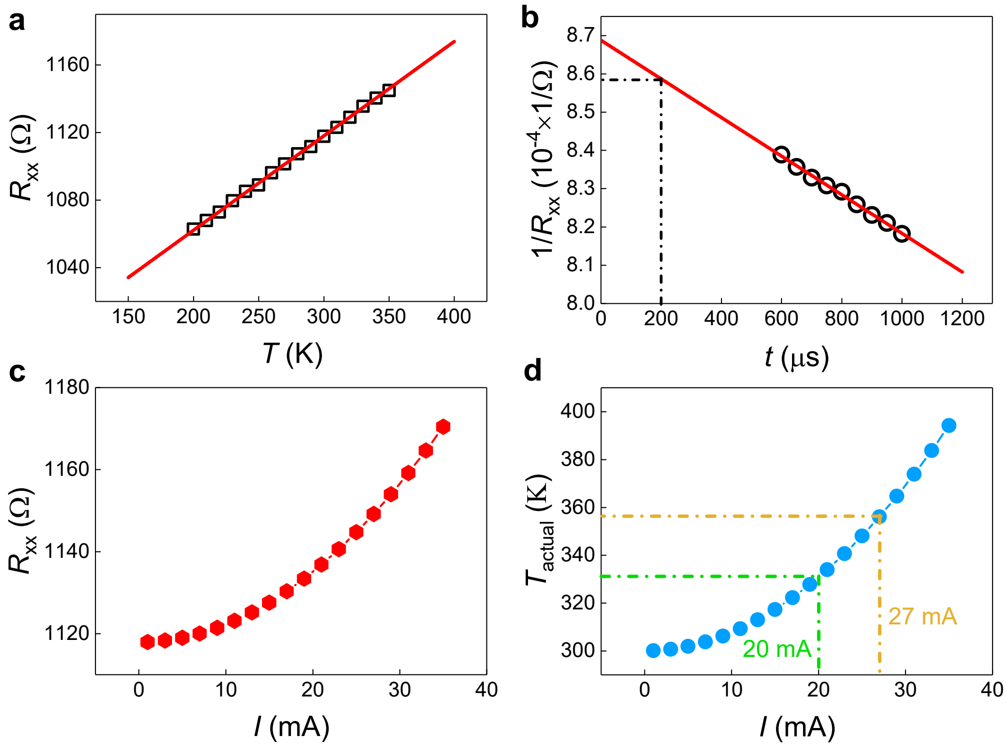

Fig. S10 a The resistance R_{xx} of the $Mn_3Pt(5)/Ti(3)/CoFeB(1)/MgO(2)/SiO_2(2)$ device as a function of the temperature. The red line is a linear fit. **b** The pulse width dependence of the conductance of the $Mn_3Pt(5)/Ti(3)/CoFeB(1)/MgO(2)/SiO_2(2)$ device. The red line is a linear fit. **c** The resistance of the $Mn_3Pt(5)/Ti(3)/CoFeB(1)/MgO(2)/SiO_2(2)$ device versus the pulse current amplitude. (Current pulse width = 200 μs). **d** The actual device temperature with different pulse amplitudes. (Current pulse width = 200 μs) .

To determine the actual temperature of the device during the loop shift and magnetization switching measurements, we first measured the temperature dependence of the longitudinal resistance R_{xx} under a small DC current I ~ 0.1 mA. The actual temperature T of the device can be estimated by the change of R_{xx} based on the R_{xx} versus T curve in Fig. S10a.

Since the resistance R_{xx} of our $Mn_3Pt(5)/Ti(3)/CoFeB(1)/MgO(2)/SiO_2(2)$ device is in the range of kΩ, the measurement range of the voltage meter needs to be adjusted to 100 V during the measurement, and the pulse width is required to be larger than 600 μs in the resistance measurements. In contrast, we use a pulse width of 200 μs

in the loop shift and magnetization switching measurements. To compare the results in different measurements with different pulse widths, we plot R_{xx} as a function of the pulse width in Fig. S10b. The data is shown to follow the empirical formula $R_{xx} = \frac{\beta}{\alpha - I^2 \times t}$, where I and t are the pulse current magnitude and pulse width, respectively, and α and β are constant fitting parameters[19]. By extrapolating the linear fit of $1/R_{xx}$ to a pulse width of 200 μs, we can obtain the resistance R_{xx} with 200 μs pulse width. We plot R_{xx} versus I in Fig. S10c and estimate the actual temperature with each pulse current by converting R_{xx} into actual temperature as shown in Fig. S10d. Based on these measurements, we estimate the actual device temperature to be ~331 K for the maximum current in the AHE loop shift measurement (Fig. 3 of the manuscript) and ~356 K for the maximum current in the magnetization switching measurement (Figs. 4c and 4d of the manuscript), which are well below the T_N of Mn_3Pt (~475 K).

Note 11. Field-free current-induced magnetization switching at different ϕ_E

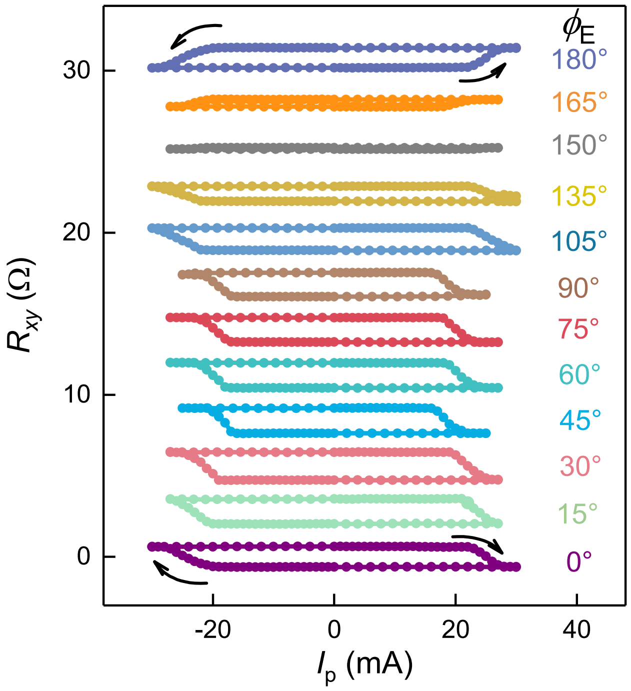

Fig. S11 Field-free current-induced magnetization switching of the $Mn_3Pt/Ti/CoFeB/MgO/SiO_2$ heterostructure for different ϕ_E.

Figure S11 shows the current-induced magnetization switching of the $Mn_3Pt/Ti/CoFeB/MgO/SiO_2$ heterostructure without the assistance of a magnetic field for ϕ_E ranging from 0° to 180°. As shown in Fig. S11, for the Hall bar with different ϕ_E, the switching ratio and switching polarity vary with ϕ_E. The field-free current-induced magnetization switching achieved illustrates the existence of σ_{zx}^z and its associated out-of-plane SOT in the $Mn_3Pt/Ti/CoFeB/MgO/SiO_2$ heterostructure. It is seen that the switching polarity for ϕ_E less than 150° is clockwise, whereas for ϕ_E from 150° to 180° it is anticlockwise. The behavior of the field-free current-induced magnetization switching for different ϕ_E can be attributed to the σ_{zx}^z induced spin torque, which is consistent with the trend of H_{eff}^z for different ϕ_E.

Note 12. The AHE loops in the Mn_3Pt/Ti/CoFeB/MgO/SiO_2 heterostructure for different premagnetization field

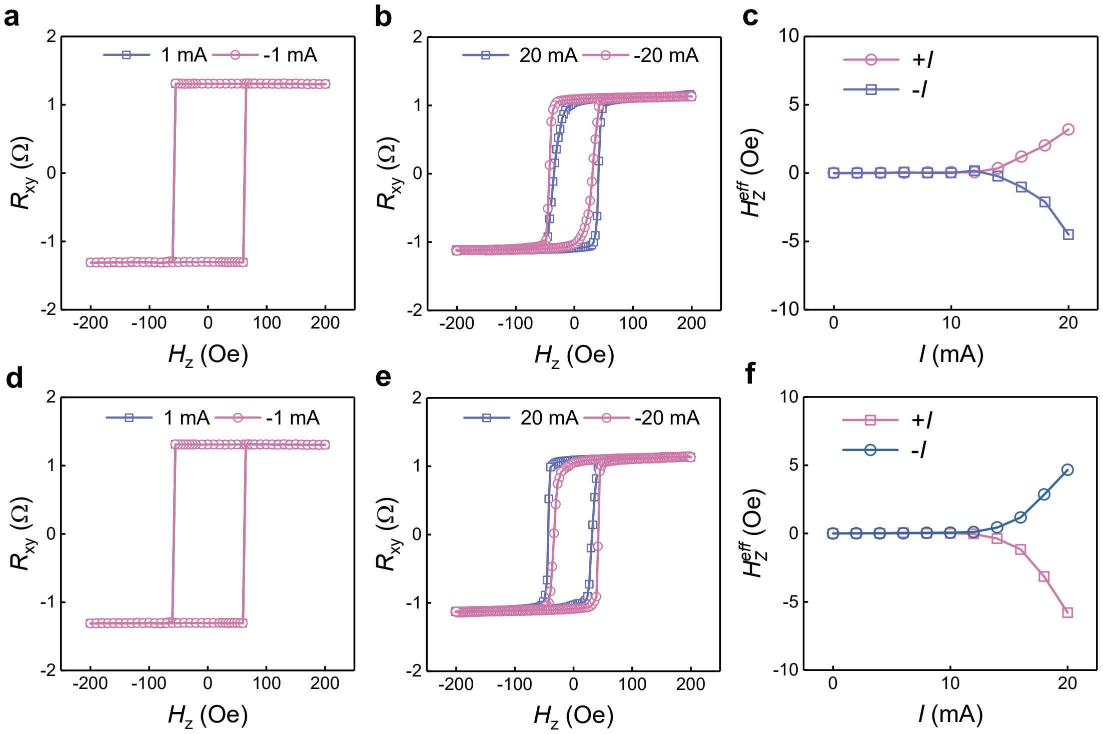

Fig. S12 a-c The AHE loops under $I = \pm 1$ mA, ±20 mA, and the I dependence of H_z^{eff} at selected $\phi_{\mathrm{E}} = 0°$ with a premagnetization field of 8 T along the [001] direction. **d-f** Similar to (**a-c)** but with a premagnetization field of 8 T along the $[00\bar{1}]$ direction.

Figure S12 shows the loop shift measurements for $\phi_{\mathrm{E}} = 0°$ with the application of a premagnetization field H_{pre} of 8 T along the [001] and $[00\bar{1}]$ direction. The expected polarity reversal is indeed observed between loop shift measurements with an H_{pre} of 8 T along the [001] and $[00\bar{1}]$ directions, consistent with the magnetization switching measurements and corroborates again the presence of the $\mathcal{T}$-odd spin Hall effect, i.e., the magnetic spin Hall effect, and the presence of the nonvanishing net magnetization.

References

1. Zelezny, J., Zhang, Y., Felser, C. & Yan, B. Spin-Polarized Current in Noncollinear Antiferromagnets. *Phys. Rev. Lett.* **119**, 187204 (2017).
2. Bai, H. et al. Control of spin-orbit torques through magnetic symmetry in differently oriented noncollinear antiferromagnetic Mn_3Pt. *Phys. Rev. B* **104**, 104401 (2021).
3. Liu, L., Moriyama, T., Ralph, D. C. & Buhrman, R. A. Spin-torque ferromagnetic resonance induced by the spin Hall effect. *Phys. Rev. Lett.* **106**, 036601 (2011).
4. Mecking, N., Gui, Y. S. & Hu, C. M. Microwave photovoltage and photoresistance effects in ferromagnetic microstrips. *Phys. Rev. B* **76**, 224430 (2007).
5. Kubota, H. et al. Quantitative measurement of voltage dependence of spin-transfer torque in MgO-based magnetic tunnel junctions. *Nat. Phys.* **4**, 37-41 (2007).
6. Mellnik, A. R. et al. Spin-transfer torque generated by a topological insulator. *Nature* **511**, 449-451 (2014).
7. Garello, K. et al. Symmetry and magnitude of spin–orbit torques in ferromagnetic heterostructures. *Nat. Nanotechnol.* **8**, 587-593 (2013).
8. MacNeill, D. et al. Control of spin–orbit torques through crystal symmetry in WTe_2/ferromagnet bilayers. *Nat. Phys.* **13**, 300-305 (2016).
9. Zhou, J. et al. Magnetic asymmetry induced anomalous spin-orbit torque in IrMn. *Phys. Rev. B* **101**, 184403 (2020).
10. Nan, T. et al. Controlling spin current polarization through non-collinear antiferromagnetism. *Nat. Commun.* **11**, 4671 (2020).
11. You, Y. et al. Cluster magnetic octupole induced out-of-plane spin polarization in antiperovskite antiferromagnet. *Nat. Commun.* **12**, 6524 (2021).
12. Bai, H. et al. Observation of Spin Splitting Torque in a Collinear Antiferromagnet RuO_2. *Phys. Rev. Lett.* **128**, 197202 (2022).
13. Bose, A. et al. Tilted spin current generated by the collinear antiferromagnet ruthenium dioxide. *Nat. Electron.* **5**, 267-274 (2022).
14. Avci, C. O. et al. Interplay of spin-orbit torque and thermoelectric effects in ferromagnet/normal-metal bilayers. *Phys. Rev. B* **90**, 224427 (2014).
15. Hayashi, M., Kim, J., Yamanouchi, M. & Ohno, H. Quantitative characterization of the spin-orbit torque using harmonic Hall voltage measurements. *Phys. Rev. B* **89**, 144425 (2014).
16. Roschewsky, N. et al. Spin-orbit torque and Nernst effect in Bi-Sb/Co heterostructures. *Phys. Rev. B* **99**, 195103 (2019).
17. MacNeill, D. et al. Thickness dependence of spin-orbit torques generated by WTe_2. *Phys. Rev. B* **96**, 054450 (2017).
18. Liu, Z. Q. et al. Electrical switching of the topological anomalous Hall effect in a non-collinear antiferromagnet above room temperature. *Nat. Electron.* **1**, 172-177 (2018).
19. Liu, L. et al. Electrical switching of perpendicular magnetization in a single

ferromagnetic layer. *Phys. Rev. B* **101**, 220402(R) (2020).
